# Cross-sectional and longitudinal risk of physical impairment in a cohort of postmenopausal women who experience physical and verbal abuse

**DOI:** 10.1186/s12905-015-0258-2

**Published:** 2015-11-11

**Authors:** M. Brad Cannell, Julie C. Weitlauf, Lorena Garcia, Elena M. Andresen, Karen L. Margolis, Todd M. Manini

**Affiliations:** Department of Biostatistics and Epidemiology, University of North Texas Health Science Center, Fort Worth, TX USA; Veterans Affairs Palo Alto Health Care System, Palo Alto, CA USA; Department of Psychiatry and Behavioral Sciences, Stanford University School of Medicine, Stanford, CA USA; Department of Public Health Sciences, University of California Davis, Davis, CA USA; School of Public Health, Oregon Health & Science University, Portland State University, Portland, OR USA; HealthPartners Institute for Education and Research, Minneapolis, MN USA; Department of Aging and Geriatric Research, University of Florida, Gainesville, FL USA

**Keywords:** Violence, Women, Physical function, WHI, Disability

## Abstract

**Background:**

Exposure to interpersonal violence, namely verbal and physical abuse, is a highly prevalent threat to women’s health and well-being. Among older, post-menopausal women, several researchers have characterized a possible bi-directional relationship of abuse exposure and diminished physical functioning. However, studies that prospectively examine the relationship between interpersonal abuse exposure and physical functioning across multiple years of observation are lacking. To address this literature gap, we prospectively evaluate the association between abuse exposure and physical functioning in a large, national cohort of post-menopausal women across 12 years of follow-up observation.

**Methods:**

Multivariable logistic regression was used to measure the adjusted association between experiencing abuse and physical function score at baseline in 154,902 Women’s Health Initiative (WHI) participants. Multilevel modeling, where the trajectories of decline in physical function were modeled as a function of time-varying abuse exposure, was used to evaluate the contribution of abuse to trajectories of physical function scores over time.

**Result:**

Abuse was prevalent among WHI participants, with 11 % of our study population reporting baseline exposure. Verbal abuse was the most commonly reported abuse type (10 %), followed by combined physical and verbal abuse (1 %), followed by physical abuse in the absence of verbal abuse (0.2 %). Abuse exposure (all types) was associated with diminished physical functioning, with women exposed to combined physical and verbal abuse presenting baseline physical functioning scores consistent with non-abused women 20 years senior. Results did not reveal a differential rate of decline over time in physical functioning based on abuse exposure.

**Conclusions:**

Taken together, our findings suggest a need for increased awareness of the prevalence of abuse exposure among postmenopausal women; they also underscore the importance of clinician’s vigilance in their efforts toward the prevention, early detection and effective intervention with abuse exposure, including verbal abuse exposure, in post-menopausal women. Given our findings related to abuse exposure and women’s diminished physical functioning at WHI baseline, our work illuminates a need for further study, particularly the investigation of this association in younger, pre-menopausal women so that the temporal ordering if this relationship may be better understood.

## Background

Interpersonal abuse exposure is a significant public health threat for women [1–31]. While the disproportionate risk of young women (e.g., of childbearing age), particularly those who are pregnant, is well documented [1–16], a growing empirical literature also characterizes the significant prevalence and public health implications of interpersonal abuse exposure, including verbal, physical or sexual abuse, neglect, and financial exploitation, among older, post-menopausal women [17–32]. For example, Zink et al. [33] conducted a telephone survey investigating prevalence of abuse exposure among more than 3500 women over age 55. They write that 1.52 % of their study participants reported physical abuse in their intimate relationships since age 55, and that 0.41 % reported physical abuse exposure, 1.12 % reported sexual abuse exposure, and 1.62 % were threatened with physical harm by their partner in the past year [34]. Similarly, Bonomi found lifetime prevalence of physical, verbal or sexual abuse among women over age 65 to be 26.5 %, with 2.2 % reporting abuse in the past year [23]. Further, Amstadter reported a past-year prevalence of emotional abuse among older women of about 4.6 %, as well as a past-year prevalence of physical abuse among older women of about 1.6 % [35].

As reports of abuse perpetrated against older, post-menopausal women have increased over the prior decade, research on risk factors and health/functional consequences of abuse exposure has correspondingly proliferated [21, 22, 24–26, 29, 30]. Linkages between verbal and physical abuse exposure, and increased risk of all-cause mortality among older women, have been reported in several prior studies [21, 22], as has evidence of bi-directional relationships between verbal and physical abuse exposure and diminished physical and mental health [29]. A bi-directional relationship between abuse exposure and diminished physical functioning in older women [36–38] has also been proposed, with some prior work characterizing physical functioning as an important risk factor for verbal and/or physical abuse in this population [36–38], and other work suggesting that abuse among older women may degrade physical functioning, ultimately leading to disability [22].

The research to date provides a strong foundation; however, gaps in our understanding of the relationship between abuse exposure and physical function remain. Specifically, the relationship is inconsistent across studies - others have found no association between abuse exposure and physical function [39]. Moreover, the preponderance of studies using cross-sectional research designs, which are unable to investigate the temporal ordering of abuse exposure and diminished physical functioning, have prevented a complete understanding of this association. Prospective studies that examine the association between abuse exposure and trajectories of decline in physical functioning over multi-year periods are warranted, and could deepen our understanding of this relationship. Indeed, such work could set the stage for the evaluation of mechanistic questions about this association, including whether declines in physical functioning mediate the association between abuse exposure and poor physical health, mental health and mortality risk.

As a first step towards that goal, the present work investigates the association between physical and verbal abuse and physical function in a large cohort of postmenopausal women, aged 50–79 (at baseline), who participated in the Women’s Health Initiative (WHI). We hypothesized that women who experienced physical and/or verbal abuse in the year prior to baseline would have lower levels of physical function at baseline compared to women who did not experience abuse. In addition, we hypothesized that women with baseline abuse exposure would have a greater rate of decline in physical functioning over time. To test these hypotheses, we evaluated the relationship between abuse exposure and physical functioning at baseline, and prospectively evaluated the subsequent rate of decline in physical function over an average of 12 years of follow-up.

## Methods

### Participants

The Women’s Health Initiative is a large, multicenter study sponsored by the National Heart, Lung, and Blood Institute (NHLBI) designed to evaluate women’s post-menopausal risk for heart disease, cancer, and osteoporotic fractures [40–42]. A complete description of the WHI methodology (including recruitment procedures) is published elsewhere [40–43]. WHI consisted of two main components: a clinical trial (CT), and an observational study (OS). Beginning in September 1993 postmenopausal women, aged 50 to 79, were recruited at 40 clinical centers in the United States using mass mailings derived from voter registration lists, vehicle registration lists, and driver’s license lists. Baseline measurements occurred between 1993 and 1998. Women enrolled in the WHI completed clinical interviews in person, face-to-face clinical assessments, and completed a series of self-report surveys designed to gather additional information about their medical and reproductive health history, medication use, health risks, including exposure to verbal or physical abuse in the 12 months prior to study baseline, health related behaviors and other lifestyle factors (e.g., smoking, physical activity), and psychosocial functioning and quality of life at baseline. Follow-up assessments included annual, mailed questionnaires about health and functioning, and regularly scheduled study-related physical health examinations. Health and mortality outcomes were locally and centrally adjudicated, the methods of which are fully delineated in Curb et al. [40, 43, 44].

**Fig. 1:**
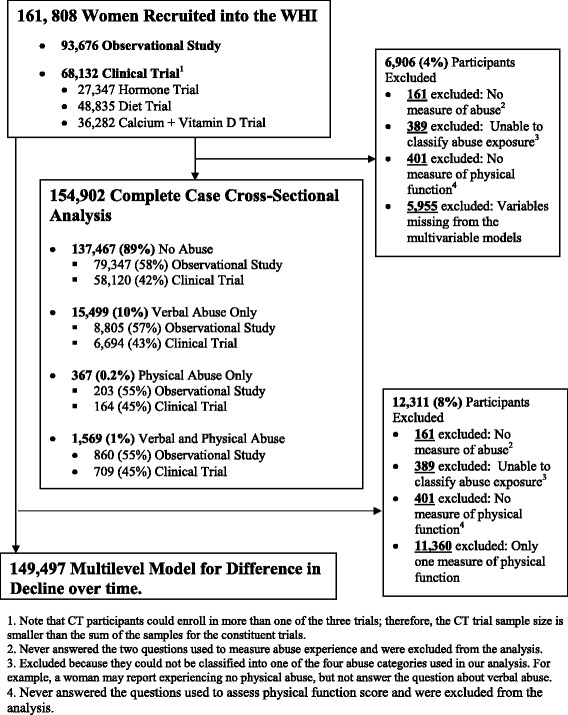
Included participants

Of the 161,808 women enrolled in WHI, 161 (0.1 %) never answered the two questions used to measure abuse experience, 389 (0.2 %) could not be classified into one of the four abuse categories used in our analysis, 401 (0.2 %) women had no physical function score, and 5955 (4 %) women were excluded because they were missing data at all follow-up occasions for covariates of interest. Excluded women were older, less likely to be white non-Hispanic, less likely to be married, had lower income, and were in poorer health than those included in our study. Notably, there was no difference in the probability of experiencing abuse, and removal of these women did not significantly change the unadjusted coefficient for the effect of abuse on physical function. These exclusions led to a final sample size for the current analysis of 154,902 women (See Fig. 1).

### Exposure

The primary exposure of interest was self-reported physical and verbal abuse. Women participating in the WHI CT were asked two questions pertaining to abuse at baseline and their year 1 follow-up. An 8.6 % subsample of the women in the menopausal hormone therapy arm and a 4.3 % subsample of the women in the dietary modification arm were asked again at years 3, 6, and 9. Women participating in the WHI OS were asked the same two abuse questions at baseline and again in year 7. A subsample of women who were enrolled in the WHI extension studies were asked again in years 1–10 of the extension study (12–21 years after the study recruitment began). The two abuse questions come from previous studies by Matthews and colleagues [45], and are worded as follows: “Over the past year: Were you physically abused by being hit, slapped, pushed, shoved, punched, or threatened with a weapon by a family member or close friend?” and “Over the past year: Were you verbally abused by being made fun of, severely criticized, told you were a stupid or worthless person, or threatened with harm to yourself, your possessions, or your pets, by a family member or close friend?” Based on their answers women were categorized into one of four groups: (1) No abuse, (2) verbal abuse only, (3) physical abuse only, and (4) verbal and physical abuse. This conceptualization of abuse exposure is consistent with previous studies conducted using data from the WHI [21, 29].

### Outcomes of interest

The primary outcome of interest for the current study was physical function as measured by the physical function scale (PFS) on the Rand 36-Item Health Survey (SF-36), where higher scores indicate more favorable physical function [46]. The PFS was previously used for research in many different populations, including community dwelling older adults, and found to be valid and reliable [47]. In brief, women are asked if their health limits their ability to engage in 10 different activities ranging from vigorous physical activity to bathing and dressing, and if so, by how much. Possible Responses are: “No, not limited at all”, (scored as 100); “Yes, limited a little”, (scored as 50); or “Yes, limited a lot”, (scored as 0). The scores from the ten individual questions are then averaged together resulting in a composite physical function score that ranges from 0 to 100, where 100 indicates higher levels of functioning. PFS can be evaluated as a continuous variable, where some research suggests that a 5 point change is clinically meaningful [48, 49]. Additionally, a PFS score less than or equal to 80 is used to classify one as having significant physical impairment (SPI), and is the cut point we applied in the logistic regression analysis detailed below [50].

Women participating in the WHI CT completed the PFS at baseline and their year 1 follow-up. An 8.6 % subsample of the women in the menopausal hormone therapy arm and a 4.3 % subsample of the women in the dietary modification arm completed it again at years 3, 6, and 9. Women participating in the WHI OS completed the PFS at baseline. A subsample of women who were enrolled in the WHI extension studies were asked again in years 1–10 of the extension study (12–21 years after the study recruitment began).

### Covariates

In order to isolate the effect of abuse on physical function, covariates of interest, including socio-demographic characteristics (e.g., age, race/ethnicity, marital status, income, education, living alone) physical and mental health functioning (self-reported general health, depression, smoking status, heavy drinking, social support, body mass index, self-reported general health) and WHI study assignment, were selected based on previously published literature demonstrating their association with abuse [20, 21, 25, 29, 37, 51–57] and physical function [58–76].

General health factors and health behaviors were assessed using standard questionnaires and in-person clinic interviews, clinical measurements were completed during baseline physical examinations [40].

Body Mass Index (BMI) was calculated from a participant’s height and weight and categorized according to recommendations from the National Institutes of Health [77].

Heavy alcohol use was a self-reported measure of the average number of drinks per day/week consumed over the previous 3 months. In accordance with definitions used by the Centers for Disease Control and Prevention [78] women were considered to drink heavily if they self-reported consuming more than 1 drink per day on average.

Social support was measured using nine items from the Medical Outcomes Study (range 9 to 45), with higher scores indicating more social support [79].

Depressive symptoms were assessed using the CES-D/DIS depression screener, which consists of 6 items from the Center for Epidemiologic Studies Depression Scale (CES-D) and two items from the Diagnostic Interview Schedule (DIS). Possible scores range from 0 to 1 and higher scores indicate greater likelihood of depression. A score greater than or equal to 0.06 indicated depressive disorder [80].

### Analysis

In order to investigate the relationship between abuse and physical function as robustly as possible, we conducted three distinct analyses: (1) we characterized the study population with respect to baseline sociodemographic and health factors at WHI baseline by abuse status (Table 1), and then compared baseline physical function scores by age and abuse status (Table 2); (2) we next utilized logistic regression to determine the association of baseline abuse exposure and women’s odds of significant impairment in physical functioning, defined as a physical function score < = 80 [50], at WHI baseline; and (3) we used baseline data and follow-up data about abuse status and physical function to longitudinally compare women’s physical function *trajectory* (i.e., improving, stable, or worsening) by abuse experience. Each analysis is further detailed below.

**Table 1:** Baseline characteristics of 154,902 women from the Women’s Health Initiative, by abuse experience^a^

Characteristic	*n* (%)			
	No abuse (*n* = 137,467)	Verbal abuse only (*n* = 15,499)	Physical abuse only (*n* = 367)	Verbal and physical abuse (*n* = 1569)
Age				
50–59	44,438 (32)	6376 (41)	141 (38)	774 (49)
60–69	62,243 (45)	6675 (43)	168 (46)	592 (38)
70–79	30,786 (22)	2448 (16)	58 (16)	203 (13)
Race/Ethnicity				
White, non-Hispanic	115,050 (84)	12,962 (84)	221 (60)	1062 (68)
Black, non-Hispanic	11,948 (9)	1134 (7)	86 (23)	243 (15)
Hispanic	4805 (4)	752 (5)	31 (8)	158 (10)
Other race, non-Hispanic	5664 (4)	651 (4)	29 (8)	106 (7)
Marital status				
Married or marriage-like relationship	91,792 (67)	11,003 (71)	218 (60)	982 (63)
Annual household income				
Less than $20,000	19,775 (15)	2507 (17)	99 (28)	462 (31)
$20,000–$34,999	30,318 (23)	3389 (23)	79 (22)	353 (23)
$35,000–$49,999	25,830 (19)	2891 (19)	67 (19)	232 (15)
$50,000–$74,999	25,146 (19)	2781 (19)	48 (14)	204 (13)
$75,00 or more	23,848 (18)	2459 (16)	37 (10)	158 (10)
Missing	8424 (6)	969 (6)	27 (8)	106 (7)
Education				
Less than High School	6792 (5)	754 (5)	63 (17)	170 (11)
High School Graduate	23,931 (17)	2332 (15)	54 (15)	246 (16)
Attended College	51,725 (38)	6236 (40)	143 (39)	704 (45)
College Graduate	55,019 (40)	6177 (40)	107 (29)	449 (29)
Living alone	35,053 (26)	3088 (20)	92 (25)	376 (24)
Study arm				
Clinical trial	58,120 (42)	6694 (43)	164 (45)	709 (45)
Observational study	79,347 (58)	8805 (57)	203 (55)	860 (55)
Current smoker	8522 (7)	1242 (9)	31 (9)	177 (12)
Heavy drinker^b^	15,410 (12)	1578 (11)	31 (9)	144 (10)
Social support construct^c^				
9–29	23,512 (17)	5596 (37)	100 (28)	703 (46)
30–34	20,975 (15)	3204 (21)	79 (22)	280 (18)
35–39	35,362 (26)	3437 (22)	93 (26)	292 (19)
40–43	26,709 (20)	1850 (12)	53 (15)	152 (10)
44–45	29,401 (22)	1227 (8)	38 (10)	111 (7)
Body mass index				
Neither overweight nor obese	47,313 (36)	4829 (32)	92 (26)	405 (27)
Overweight	46,164 (35)	5097 (34)	121 (35)	505 (34)
Obese I	24,125 (18)	2992 (20)	82 (23)	350 (23)
Obese II	9811 (7)	1307 (9)	35 (10)	160 (11)
Extreme obese	5069 (4)	710 (5)	19 (5)	85 (6)
General health				
Excellent or very good	81,655 (60)	7731 (50)	164 (45)	638 (41)
Depression	12,054 (9)	3816 (25)	81 (23)	588 (39)

^a^The difference in distributions between abuse exposure groups was statistically significant (*p* < 0.05) for every characteristic. The statistical significance of differences in proportions was determined using the chi-squared method

^b^In accordance with definitions used by the Centers for Disease Control and Prevention [78], women were considered to drink heavily if they self-reported consuming more than 1 drink per day on average

^c^Range 9–45; higher score indicates greater support

**Table 2:** Mean initial physical function score^a^ by baseline age group and abuse experience^b^

	No abuse		Verbal abuse only		Physical abuse only		Verbal and physical abuse	
	*n*	Mean (sd)	*n*	Mean (sd)	*n*	Mean (sd)	*n*	Mean (sd)
Age 50–59	44,438	86.0 (17.9)	6376	82.1 (20.3)	141	79.9 (21.6)	774	75.4 (25.0)
Age 60–69	62,243	81.6 (19.2)	6675	77.4 (21.3)	168	71.2 (25.2)	592	71.3 (24.8)
Age 70–79	30,786	75.1 (21.3)	2448	71.1 (22.4)	58	67.8 (24.3)	203	69.0 (23.5)

^a^Score on the physical function scale (PFS) of the Rand 36-Item Health Survey (SF-36) the first time it was administered. Scores range from 0 to 100 with higher scores indicating better physical function

^b^Self-reported abuse exposure (none, verbal only, physical only, both) the first time asked

Baseline descriptive statistics including simple bivariate analyses evaluated the association of past year abuse status with a host of background demographic and health risk variables. The statistical significance of differences in proportions was determined using the chi-squared method. It should be noted that because of our large sample size, modest absolute differences between groups are often statistically significant. We caution the reader against misinterpreting statistical significance as necessarily equating to clinical significance.

Next, baseline mean physical function scores by age group and abuse experience were calculated. Then, simple logistic regression was used to measure the unadjusted cross-sectional association between experiencing abuse and significant physical impairment at baseline. Finally, multivariable logistic regression was used to measure the adjusted odds of abuse exposure among women with significant physical impairment at baseline, compared to women without significant physical impairment at baseline, along with associated 95 % confidence intervals (Table 3).

**Table 3:** Model estimated unadjusted and adjusted odds of significant physical impairment at baseline by abuse experience at baseline

Abuse exposure	Unadjusted model (95 % CI)	Minimally adjusted model^a^ (95 % CI)	Saturated model^b^ (95 % CI)
No abuse	Referent	Referent	Referent
Verbal abuse only	1.32 (1.28, 1.37)	1.20 (1.16, 1.25)	1.16 (1.11, 1.21)
Physical abuse only	1.75 (1.43, 2.15)	1.55 (1.23, 1.95)	1.38 (1.06, 1.80)
Verbal and physical abuse	1.87 (1.69, 2.06)	1.53 (1.37, 1.72)	1.33 (1.17, 1.52)

^a^Adjusted for age, self-reported general health, and depression

^b^Adjusted for age, race/ethnicity, marital status, income, education, living alone, study arm, smoking status, heavy drinking, social support, body mass index, self-reported general health, and depression

To evaluate the longitudinal association of abuse exposure on women’s trajectory of physical function over time, we used multilevel modeling techniques where the trajectories of declines were modeled as an interaction of abuse exposure and time (years). In the multilevel models only, abuse was treated as a time varying exposure—meaning that a women’s abuse status could change over time. Specifically, women who did not report baseline abuse, but subsequently reported abuse during follow-up would be included in the “abuse exposure” from that point forward.

All analyses were conducted using Stata/MP 13.1 (StataCorp, College Station, TX). All participants gave written informed consent to participate in the study, which was overseen by the Institutional Review Boards (IRBs) at each of the 40 field centers and the Clinical Coordinating Center. The University of Florida’s IRB approved the use of de-identified data to conduct this analysis.

## Results

At baseline, 17,435 (11 %) of women reported experiencing any abuse in the past year. Fifteen thousand four hundred ninety nine women (10 %) reported experiencing verbal abuse only, 367 women (0.2 %) reported experiencing physical abuse only, and 1569 (1 %) reported experiencing both verbal and physical abuse.

Table 1 presents data related to the various key sociodemographic and health characteristics of WHI participants at baseline. As shown, women who experienced abuse were more likely to be in the youngest age group (50–59), have a lower annual household income, lower levels of social support, poorer self-reported health and more depressive symptoms than women who did not experience abuse. We again caution the reader to interpret *p*-values conservatively because of large sample sizes.

Table 2 shows the unadjusted mean physical function scores at baseline by age group and abuse experience. Within each abuse experience category we observed the expected inverse relationship between age and initial mean physical functions scores. Additionally, within each age group initial mean physical function scores tended to be progressively lower among women who had experienced verbal, physical, or both forms of abuse. For example, among women who were between the ages of 50 and 59 at baseline, those who did not experience abuse had a mean PFS of 86, those who experienced verbal abuse only had a mean PFS of 82, those who experienced physical abuse only had a mean PFS of 80, and those who experienced verbal and physical abuse had a mean PFS of 75.

Table 3 presents the results of modeling odds of significant physical impairment at baseline by type of abuse exposure, reported at baseline, using multivariable linear regression. Baseline odds of significant physical impairment were greater in women reporting baseline abuse (all types) relative to those reporting no baseline exposure. Additionally, odds of significant physical impairment were generally greater as women experienced physical abuse. However, the odds ratios were attenuated after adjustment for covariates. In the adjusted model women who experienced verbal abuse only had 1.16 times greater odds of baseline significant physical impairment than women who did not experience abuse, women who experienced physical abuse only had 1.38 times greater odds of baseline significant physical impairment than women who did not experience abuse, and women who experienced verbal and physical abuse had 1.33 times greater odds of baseline significant physical impairment than women who did not experience abuse.

Finally, Table 4 presents the results of fitting unadjusted multilevel models separately in each baseline age group to estimate the differences in rate of change (slope) in PFS between abuse categories over time. In general, trajectories of decline did not meaningfully differ by abuse experience. Complete results are shown in Table 4.

**Table 4:** Model estimated differences in physical function decline over time (in years) by age group at baseline and abuse experience^a^

	50–59 (*n* = 49,911)	60–69 (*n* = 67,702)	70–79 (*n* = 31,884)
Difference in rate of change^b^ (se)			
No abuse	Ref.	Ref.	Ref.
Verbal abuse only	−0.09 (0.02)^**^	−0.08 (0.02)^**^	0.00 (0.04)
Physical abuse only	−0.11 (0.11)	0.02 (0.12)	−0.48 (0.25)
Verbal and physical abuse	−0.11 (0.05)^*^	0.03 (0.06)	0.22 (0.14)

^*^ *p* < .05; ^**^ *p* < .001 for the null hypothesis that the difference in the current estimate and the estimate for no abuse is equal to zero

^a^Abuse exposure is modeled as a time-varying exposure in multilevel models

^b^Difference in rate of change estimates are additionally adjusted for baseline PFS

## Discussion

Results of the present study of 154,902 postmenopausal women participants in WHI reveal an 11 % 1 year prevalence of abuse exposure among middle aged and older adult women, as well as a statistically and clinically significant cross-sectional association between abuse exposure and diminished physical functioning assessed at study baseline. Verbal abuse in the absence of physical abuse (10 %) was the most prevalent exposure, combined physical and verbal was the next most common (1 %), followed by exposure to physical abuse in the absence of verbal abuse (0.2 %) (Table 1). Baseline exposure to abuse (all types) was linked to lower baseline physical functioning at baseline. However, women exposed to physical abuse—either in isolation or in combination with verbal abuse—evidenced the most pronounced deficiencies. Indeed, self-reported baseline physical functioning scores among women in this group were similar to those of non-abused women who were two decades older (see Table 2). Exposure to a single form of abuse, either verbal or physical, was also linked with lower baseline physical functioning; however, these deficiencies in physical functioning were less pronounced. Specifically, women reporting exposure to physical or verbal abuse at baseline evidenced physical functioning scores consistent with non-abused women who were as much as 10 years their senior (Table 2), and were at increased odds of significant physical impairment—even after controlling for demographic and health risk factors (Table 3). Contrary to expectation, prospective analysis over a 12 year follow-up observation period revealed that baseline abuse exposure (verbal, physical, or combined) was not associated with a differential rate of decline in physical functioning over time.

The cross-sectional analysis of baseline data does not permit determination of the directionality of the association of abuse exposure with physical functioning, or the identification of the mechanisms underlying this important relationship. It is likely that many women reporting baseline abuse exposure had a broader or more chronic history of abuse than was captured by WHI. If so, this may have led to diminished physical functioning -- directly through abuse related injury, or indirectly as a component of more general abuse related degradation in physical health -- characterized at baseline. Given the substantially greater prevalence of verbal, as opposed to physical, abuse exposure at baseline, the latter pathway seems more likely. On the other hand, diminished physical functioning -- not associated with prior abuse exposure -- could increase women’s vulnerability to abuse. The authors posit that explanatory factors underlying the association of abuse exposure and decreased physical functioning are likely multi-faceted, and all possibilities delineated above warrant further consideration in future work. It is interesting that results did not reveal a differential rate of decline in physical functioning by abuse exposure (or type of abuse) prospectively over the follow-up observation period. This may reflect a floor effect, suggesting that pronounced or accelerated decreases in physical functioning occurred in women’s pre-menopausal years, prior to WHI enrollment.

Our findings are consistent with a growing body of empirical literature that documents the significant prevalence, particularly related to verbal abuse exposure, among older women [19, 27]. Further, they are consistent with a broad prior literature related to socio-demographic and health risk factors, including diminished physical functioning, associated with abuse exposure among older women [36–38]. Findings contrast with prior work that has revealed no association between abuse exposure and physical functioning on older women [20, 81, 82], perhaps reflecting differences in measurement, or variability in age, degree of abuse exposure and degree of physical disability present among participants in these studies.

The present work also significantly extends the extant literature base on this topic as it represents the first large scale and long-term (12 years) prospective analysis of the association between abuse exposure and physical functioning among older, post-menopausal women. Moreover, our findings, which highlight markedly age-inconsistent levels of physical functioning among the youngest group of participants (50–59 years at study entry) reporting baseline abuse, suggest that investigation of the association between women’s abuse exposure and physical functioning should occur before midlife. Investigating how abuse exposure impacts physical functioning, and vice-versa, over the entire life course may help deepen our understanding of this association, and illuminate critical windows for early detection and intervention.

Limitations of our study include the fact that we could not account for lifetime abuse exposure, and thus our understanding of the temporal ordering of these exposures, or how they interacted prior to baseline remains incomplete. In addition, this data offers no contextual information about the frequency or severity of baseline verbal or physical abuse exposure among participants. As such, questions about how frequency and severity of abuse may impact physical functioning over time are beyond the scope of this paper. Additionally, we are unable to report on the relationship between women in our study who experienced abuse, and those who perpetrated abuse against them. Such information would have been valuable in deepening our understanding of risk for abuse among post-menopausal women, and for program planning pertinent to prevention, early detection and intervention efforts. Further, while physical abuse and verbal abuse are highly prevalent and impactful on women’s health and functioning, other common forms of maltreatment among older women, including sexual abuse, financial exploitation and neglect, were not measured within WHI. Additionally, we are unable to account for the possibility of reporting differences related to women’s abuse history, and it may be that some women did not disclose abuse and were subsequently misclassified in our study. While we controlled for many known confounders, including health and health risk behaviors (e.g., smoking, obesity, depression, self-reported health status), statistical adjustment may not fully account for the variance associated with these factors which would be expected to degrade physical functioning over time. Moreover, other important variables, including mental health factors such as posttraumatic stress disorder, were unmeasured in WHI and thus unaccounted for within our study. Finally, generalizability may be limited as our data is not drawn from a representative sample of the U.S. population of older women. WHI participants are, on the whole, healthier, have greater social resources, and are of higher SES than the general population—this is particularly true of the women who continued to participate in follow-up visits over time.

Nevertheless, our study also has several notable strengths. Ours is among the first to prospectively investigate the effects of verbal abuse, physical abuse, and their combination (verbal and physical abuse) on trajectories of physical functioning among older, post-menopausal women. Second, this study uses a very large and diverse population of post-menopausal women from across the U.S. Finally, our study is able to evaluate the impact of type of abuse exposure (verbal, physical, or both) on physical functioning across three distinct age groups of post-menopausal women, offering a substantial contribution to the very limited extant literature on this topic.

## Conclusions

Taken together our results offer several implications for clinical care, health policy and research. First, given that 11 % of study participants reported baseline abuse exposure, our findings underscore a need for increased awareness of the prevalence and health significance of abuse exposure among health care providers who care for post-menopausal women. Efforts to increase universal screening for abuse exposure among women, including post-menopausal women who are not yet elders, have been encouraged by several health policy agencies including the Institute of Medicine who recently urged screening for intimate partner violence and sexual abuse among women of all ages [83] and the Affordable Care Act [84] which urges resources for violence related screening, counseling and care for women. As current clinical practice regarding screening for violence is guided by prior work on intimate partner violence among younger women (i.e. with particular emphasis on the need for screening during high risk periods such as pregnancy and post-partum) or on elder abuse (i.e. with particular emphasis on the needs of the oldest old, elders who are cognitively impaired or demented or vulnerable and frail [7]), additional research that identifies the best practices for violence related screening and health policy for mid-life and early older adulthood women are needed.

Additionally, our results suggest a salient association between diminished physical functioning and baseline reporting of abuse exposure, illuminating, in particular, the dramatic age-inconsistent level of physical functioning among our youngest group of women (50–59 at study baseline). Thus, an important implication of our work relates to the need for health care providers’ awareness of the salience of diminished or age-inconsistent physical functioning as a potential risk factor for abuse or marker of abuse exposure among women. Research that examines how physical functioning and abuse exposure interrelate across women’s life span may clarify our understanding of this association, illuminate important ‘critical windows’ for intervention, and better inform health policy for women of all ages.

## Appendix

### Short List of WHI Investigators

Program Office: (National Heart, Lung, and Blood Institute, Bethesda, Maryland) Jacques Rossouw, Shari Ludlam, Dale Burwen, Joan McGowan, Leslie Ford, and Nancy Geller.

Clinical Coordinating Center: Clinical Coordinating Center: (Fred Hutchinson Cancer Research Center, Seattle, WA) Garnet Anderson, Ross Prentice, Andrea LaCroix, and Charles Kooperberg.

Investigators and Academic Centers: (Brigham and Women's Hospital, Harvard Medical School, Boston, MA) JoAnn E. Manson; (MedStar Health Research Institute/Howard University, Washington, DC) Barbara V. Howard; (Stanford Prevention Research Center, Stanford, CA) Marcia L. Stefanick; (The Ohio State University, Columbus, OH) Rebecca Jackson; (University of Arizona, Tucson/Phoenix, AZ) Cynthia A. Thomson; (University at Buffalo, Buffalo, NY) Jean Wactawski-Wende; (University of Florida, Gainesville/Jacksonville, FL) Marian Limacher; (University of Iowa, Iowa City/Davenport, IA) Robert Wallace; (University of Pittsburgh, Pittsburgh, PA) Lewis Kuller; (Wake Forest University School of Medicine, Winston-Salem, NC) Sally Shumaker.

Women’s Health Initiative Memory Study: (Wake Forest University School of Medicine, Winston-Salem, NC) Sally Shumaker.

For a list of all the investigators who have contributed to WHI science, please visit: https://www.whi.org/researchers/Documents%20%20Write%20a%20Paper/WHI%20Investigator%20Long%20List.pdf.
